# A tablet-based multi-dimensional drawing system can effectively distinguish patients with amnestic MCI from healthy individuals

**DOI:** 10.1038/s41598-023-46710-y

**Published:** 2024-01-10

**Authors:** Xiaonan Zhang, Liangliang Lv, Jiani Shen, Jinyu Chen, Hui Zhang, Yang Li

**Affiliations:** 1 Department of Radiology, First Hospital of Shanxi Medical University, Taiyuan, China; 2 Department of Medical Imaging, Shanxi Medical University, Taiyuan, China; 3 Lvliang People’s Hospital, Lvliang, China; 4 Department of First Clinical Medicine, Shanxi Medical University, Taiyuan, China; 5 Shanxi Key Laboratory of Intelligent Imaging and Nanomedicine, First Hospital of Shanxi Medical University, Taiyuan, China; 6 Department of Neurology, First Hospital of Shanxi Medical University, Taiyuan, China

**Keywords:** Health care, Computational biology and bioinformatics, Predictive markers, Alzheimer's disease, Neurology, Neurodegenerative diseases

## Abstract

The population with dementia is expected to rise to 152 million in 2050 due to the aging population worldwide. Therefore, it is significant to identify and intervene in the early stage of dementia. The Rey-Osterreth complex figure (ROCF) test is a visuospatial test scale. Its scoring methods are numerous, time-consuming, and inconsistent, which is unsuitable for wide application as required by the high number of people at risk. Therefore, there is an urgent need for a rapid, objective, and sensitive digital scoring method to detect cognitive dysfunction in the early stage accurately. This study aims to clarify the organizational strategy of aMCI patients to draw complex figures through a multi-dimensional digital evaluation system. At the same time, a rapid, objective, and sensitive digital scoring method is established to replace traditional scoring. The data of 64 subjects (38 aMCI patients and 26 NC individuals) were analyzed in this study. All subjects completed the tablet's Geriatric Complex Figure (GCF) test, including copying, 3-min recall, and 20-min delayed recall, and also underwent a standardized neuropsychological test battery and classic ROCF test. Digital GCF (dGCF) variables and conventional GCF (cGCF) scores were input into the forward stepwise logistic regression model to construct classification models. Finally, ROC curves were made to visualize the difference in the diagnostic value of dGCF variables ***vs***. cGCF scores in categorizing the diagnostic groups. In 20-min delayed recall, aMCI patients' time in air and pause time were longer than NC individuals. Patients with aMCI had more short strokes and poorer ability of detail integration (all *p* < 0.05). The diagnostic sensitivity of dGCF variables for aMCI patients was 89.47%, slightly higher than cGCF scores (sensitivity: 84.21%). The diagnostic accuracy of both was comparable (dGCF: 70.3%; cGCF: 73.4%). Moreover, combining dGCF variables and cGCF scores could significantly improve the diagnostic accuracy and specificity (accuracy: 78.1%, specificity: 84.62%). At the same time, we construct the regression equations of the two models. Our study shows that dGCF equipment can quantitatively evaluate drawing performance, and its performance is comparable to the time-consuming cGCF score. The regression equation of the model we constructed can well identify patients with aMCI in clinical application. We believe this new technique can be a highly effective screening tool for patients with MCI.

## Introduction

The population with dementia is expected to rise to 152 million in 2050 due to the aging population worldwide^1^. It brings enormous mental pressure and economic burden to caregivers, the medical industry, and society. Alzheimer's disease (AD) is the most common type of dementia, accounting for about 50–60% of the dementia population in the elderly^2^. Mild cognitive impairment (MCI) is an intermediate stage between normal cognitive aging and dementia due to AD^3^. Especially individuals diagnosed with amnesia MCI (aMCI), about 10–15% of them are converted to AD every year^3^. Therefore, it is of great significance to identify and intervene early.

The Rey–Osterrieth Complex Figure (ROCF) test is widely used to assess cognitive disorders' visuo-constructional ability and visual memory, including AD^4, 5^. At present, a variety of versions and scoring methods have been developed^5^. The neuropsychological dysfunction of a subject can be assessed by drawing performance, including attention and concentration, fine-motor coordination, visuospatial perception, non-verbal memory, planning and organization, and spatial orientation^6, 7^. Conventional scoring methods of ROCF focus on quantificationally scoring the final product of drawing by assessing its elements' shape and position accuracy^8–10^. Although several process scoring methods (e.g., Bennett-Levy^11^, Boston Qualitative Scoring System^12^, and Developmental Scoring System^13^) can quantify the drawing order and direction of elements to increase the evaluation of executive functions and organizational strategies, they are time-consuming, and poor evaluation consistency and expertise are needed^5^.

In recent years, digitizing traditional cognitive scales has become a hot spot in neuroscience^14^. Initially, researchers developed automatic scoring software for the ROCF test using artificial intelligence (e.g., computer vision technology^15^, Gaussian filter method^16^, and deep machine learning algorithm^17, 18^), which can almost achieve 94% consistency with human scores. However, neither of these studies evaluated the drawing order but focused on identifying outlines and details related to traditional scoring methods. Previously, only two studies used digital devices (such as a digital pen^19^ or tablet^20, 21^) to capture the drawing process and analyzed the subjects' drawing behavior patterns by extracting spatial, procedural, and kinematic dimension parameters. Poreh et al.^19^ used a digital pen to capture the drawing process and recorded the movement of the pen to the laptop through an infrared receiver. Thereby, it realized a semi-automatic analysis of the continuity and symmetry variables in Bennett-levy scoring. Also, Kim et al.^20^used a tablet (Samsung Galaxy Book) to record the drawing process and automatically extract stroke parameters (e.g., time, speed, and length) and graphical space information (e.g., position of center and mass). It also used 2D technology to analyze the shape similarity between the original and copied figure. The results showed that AD patients copied the figure more fragmentedly with a longer pause and were more inclined to move the figure closer to the target image with lower accuracy than NC individuals (i.e., individuals with normal cognition). Late-onset AD showed signs of leftward deviation in space utilization.

It is well known that the digital parameters of the clock drawing test have successfully proved the cognitive process, and its high sensitivity and specificity in distinguishing patients with aMCI or mild AD from NC individuals are remarkable^22–26^. The behavioral pattern of drawing complex figures in aMCI patients has not yet been explored. And whether the kinematic parameters during the drawing process can distinguish aMCI patients from NC individuals for early cognitive impairment screening.

**Figure 1:**
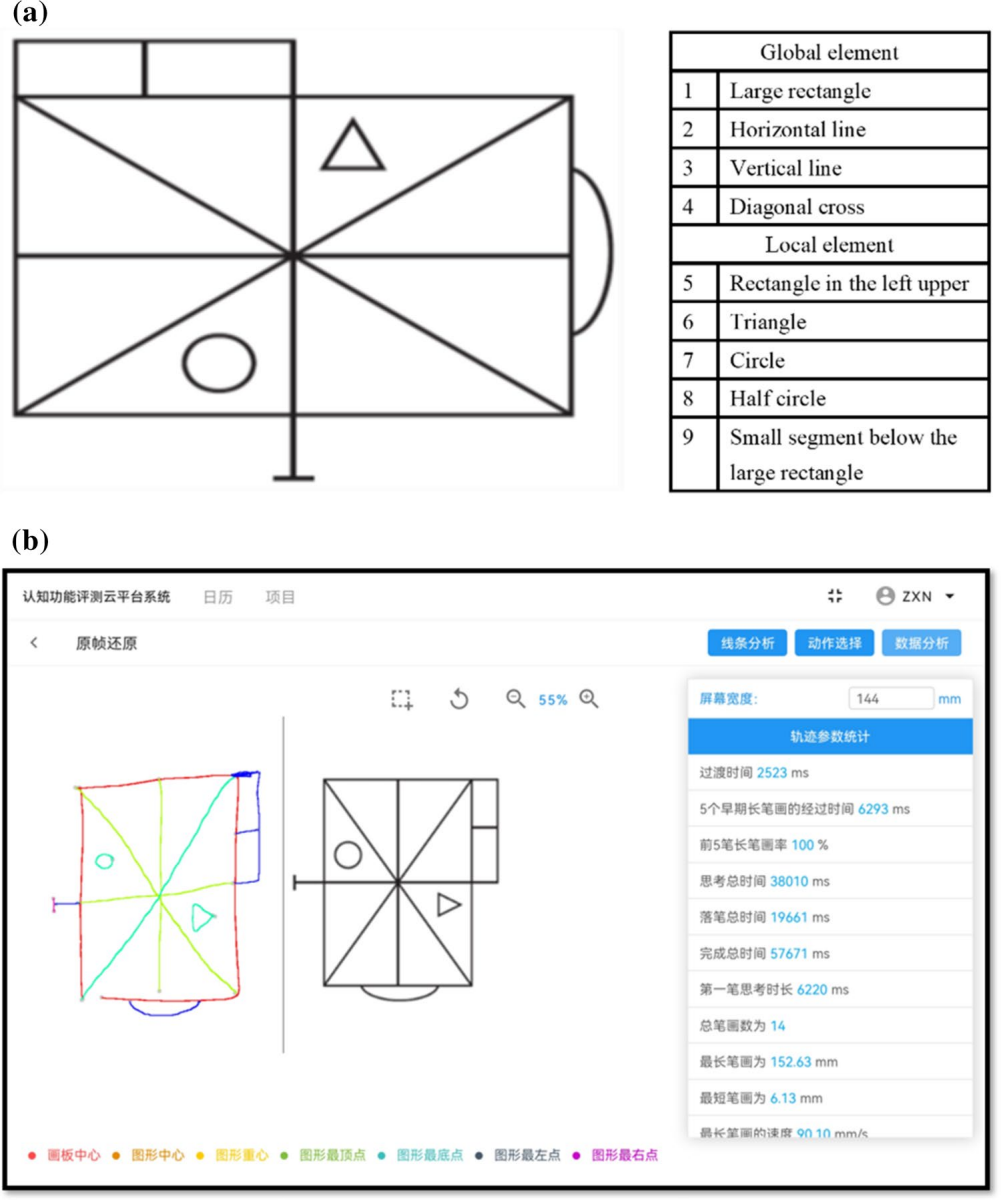
Digital Geriatric Complex Figure (dGCF) Test. (**a**) The GCF is a simplified organizational strategy model based on the classic ROCF test. It consists of four global and five local elements. (**b**) dGCF software consists of three modules: drawing area, line selection, and data analysis (size: 10.8-inch; resolution: 2560 × 1600; Huawei Tablet M6). The upper part of the tablet is defined as the display area, and the lower part is defined as the drawing area.

This study explored the drawing characteristics of aMCI patients in the copying, 3-min recall, and 20-min delayed recall of the complex figure test. Compared with traditional scores, the diagnostic value of digitized variables in distinguishing aMCI patients from NC individuals. To realize this scheme, we chose a simplified ROCF—Geriatric Complex Figure (GCF) (Fig. 1a) developed by Poreh^27^in 2002. It is an organizational strategy model based on the classic ROCF test. Its copying, recall, and strategy scores are well-distributed among healthy older people (over 60 years old), indicating that it is more suitable for the elderly than the ROCF^19^. Using the simplified figure can avoid the overlap of strokes, which is conducive to recognizing each stroke with digital equipment in a limited space. It can also improve the subjects' cooperation and enthusiasm.

This study aims to clarify the organizational strategy of aMCI patients to draw complex figures through a multi-dimensional digital evaluation system. At the same time, a rapid, objective, and sensitive digital scoring method is established to replace traditional scoring.

## Materials and methods

### Participants

Participants were recruited from the memory or neurology clinic of the First Hospital of Shanxi Medical University from November 2020 to November 2021. The study group included 38 patients with aMCI and 26 NC individuals with junior high school education or above (> 6 years). They all had normal vision and hearing and could complete neuropsychological assessment and drawing tasks. All participants underwent a rigorous evaluation, including a standardized neuropsychological battery, structural magnetic resonance imaging (MRI) of the brain, blood tests, and neurologist diagnosis. Patients with aMCI were enrolled in the study according to the National Institute on Aging and the Alzheimer's Association (NIAA) clinical MCI criteria in 2011^28^. Its core criteria include (1) memory decline for at least six months (confirmed by informants); (2) MMSE score ≥ 24, with objective evidence of impairment in one or more cognitive domains (scores adjusted for age and education were lower than 1.5SD in healthy elderly); (3) maintain the independent activities of daily living; (4) CDR score was 0.5. NC individuals should meet the following criteria: (1) no episodic memory impairment or objective evidence; (2) MMSE score ≥ 26, CDR score was 0, and intact activities of daily living. Exclusion criteria: (1) We excluded patients diagnosed with dementia (CDR > 0.5). (2) Patients who have suffered from cerebrovascular stroke have apparent symptoms or signs of neurological impairment at the onset of the disease. Cerebral structure MRI showed the corresponding responsible lesions. (3) Cerebral MRI indicated severe leukoencephalopathy (Faskass score ≥ 3). (4) Alcoholism, drug addiction, brain trauma, epilepsy, encephalitis, normal cranial pressure hydrocephalus, and other neurological diseases that can cause cognitive impairment are also excluded. (5) Systemic diseases that may lead to MCI, such as liver and kidney insufficiency, endocrine diseases, vitamin deficiency, etc. (6) Suffering from anxiety, depression, or schizophrenia.

The First Hospital of Shanxi Medical University Ethics Committee approved the study. All methods of this study were carried out in strict accordance with the Declaration of Helsinki. All subjects or their legal guardians signed informed consent after knowing the details of the study.

### Neuropsychological assessments

All subjects received a standardized neuropsychological test battery, including memory, language, attention, visuospatial, and executive function tests. Tests of interest were selected from each cognitive domain. All tests were converted to standard z-scores using published criteria, and then composite scores for each cognitive domain were created by calculating the average of each category. Mini-Mental State Examination (MMSE, Chinese version)^29, 30^, Montreal Cognitive Assessment Scale (MoCA, Chinese version)^31, 32^, and Clinical Dementia Rating (CDR)^33^were used to evaluate the general cognitive level.

Memory—Immediate and delayed recall of Auditory Verbal Learning Test (AVLT, Chinese version)^34, 35^ and ROCF test^8^. We use the 15-word Chinese version of AVLT. It is divided into five categories, each containing three words. The subjects were asked to learn three times and recall after each repetition. The presentation of words is random and does not follow semantic categories. After a 20-min interval, the subjects were asked to perform long-delayed recall, cue recall, and recognition tests. During the 20-min break, the subjects performed other non-verbal tasks. The 18 units in the ROCF test were scored separately (Osterrich scoring^8^) regarding accuracy and location. Each unit can be rewarded with 0–2 points; the raw score was 0–32.

Language—The Boston Naming Test (BNT, 30-item version)^36, 37^and Verbal Fluency Test (Animal, VFT)^38^. In the Chinese version of the BNT test, subjects were asked to name 30 pictures without a time limit, with a total score of 30. The VFT paradigm requires subjects to list as many animals as possible in one minute. We recorded the total number of animals.

Attention—Forward and Backward digit span test (DST)^39^. The evaluator reads some numbers in this test, and the subject listens carefully. When they finished reading, the subjects were asked to recite it similarly or backward. We recorded the number of correct strings completed.

Visuospatial function—Judgment of Line Orientation (JLO, 35-item version)^40^ and copying of ROCF test. The JLO scale consists of 35 cards, the first 5 for practice and the last 30 for testing. The reference card consists of 11 segments arranged in a fan pattern. The test card consists of two line segments of different lengths and directions. We recorded the number of correct lines completed, with a maximum score of 30.

Executive function—Trial Making Test (TMT, Chinese version) parts A and B^41, 42^and Stroop's color word reading test (SCWT, Chinese version)^43, 44^. In TMT-A, the subjects are asked to connect a sequence of Arabic numerals (1–25). The TMT-B contains white-circled Arabic numerals (1–13) and black-circled Arabic numerals (1–12). The subjects were asked to connect Arabic numerals in alternating order of white circles followed by black circles. We recorded the time it took to complete the test. The SCWT contains 3 subtests. The SCWT-A consists of a page of 100 color words (red, green, blue) in black font. The SCWT-B consists of a page of 100 "X" symbols in red, green, and blue font. The SCWT-C is a page of 100 words that use the word of the A subtest and the color of the B subtest (color and word do not match). The subjects read column by column as fast as they could. We recorded the number of correct words completed within 45 s of each paradigm.

### Digital geriatric complex figure test

All subjects had to complete the GCF test on the tablet (Fig. 1b), including copying, 3-min recall, and 20-min delayed recall. We define the upper part of the tablet as the display area (rendering GCF) and the lower part as the drawing area. The subjects must use a digital pen to draw in the drawing area. After 3 min and 20 min, they were asked to draw again. The total time of each drawing should be within 10 min. The trace of the figure was recorded on the tablet at a sampling frequency of 60 Hz (size: 10.8-inch; resolution: 2560 × 1600; Huawei Tablet M6).

Digital GCF equipment can extract the number, length, speed, and interval time of strokes in the drawing process and reproduce the drawing track. In addition to the element latency time, which requires the analyst to outline the first stroke of each local element manually, other parameters can be automatically analyzed by the digital equipment. The dGCF variable is defined as follows:

Transition time—the time to start drawing long strokes after short strokes in all short-long stroke sequences, s

Elapsed time of 5 early long strokes—total time of drawing 5 early long strokes, s.

First 5 stroke ratios—the proportion of long strokes in the first 5 strokes, %.

Speed of the longest stroke—the average speed of the longest stroke, cm/s.

Time in air—total time from one stroke to the next stroke, s.

Total time—the sum of time in air and time on the surface, s.

First stroke latency—time in air before drawing the first stroke, s.

Average element latency—average value of thinking time before drawing the first stroke of all local elements.

Whole area—the minimum circumscribed rectangular area of the figure, cm^2^.

In addition, the 9 units in GCF test were scored separately in terms of accuracy and location, which was consistent with Osterrich's standardized score on the ROCF test^8^. The raw score was 0–16. To avoid the learning effect between ROCF and GCF tests, we divided them into two times with an interval of one day.

### Data analysis

All statistical analyses were processed using SPSS (version 26) and GraphPad Prism (version 9) software, and a* p* value < 0.05 was considered significant. The independent sample t-test was applied to assess the statistical significance between groups for age and education level because they follow a normal distribution. The Chi-Square test was used for group differences in gender distribution. Group differences in cognitive characteristics and digital parameters were examined running the nonparametric Mann–Whitney U test because they did not follow a normal distribution.

To verify the validity of GCF data, we used Pearson correlation to compare classical ROCF with GCF test total scores. The k-means clustering was used to analyze the cut-off value of long/short strokes in dGCF software. After adjusting for age, sex, and education level, we used partial correlation to compare the dGCF parameter with the cognitive domain composite scores to determine whether the digital parameter represents a specific cognitive impairment. We chose dGCF parameters with significant differences between groups in 20-min delayed recall and cGCF scores as continuous independent variables and aMCI against NCs as dichotomous dependent variables. A combination of dGCF parameters and cGCF scores was entered into the logistic regression model using a positive stepwise inclusion model.

Finally, we calculated the Receiver Operating characteristics (ROC) curves and the area under the curves (AUC) of the logistic models to compare the diagnostic value of different models (dGCF vs. cGCF scores vs. d + cGCF) for patients in distinguishing and NC individuals.

## Results

### Demographic characteristics and neuropsychological tests between NC and aMCI groups

There was no significant difference in age, sex distribution, and education level between NC individuals and aMCI patients (*p* > 0.05). The MMSE (*p* = 0.001) and MOCA (*p* < 0.001) scores of aMCI patients were higher than those of NC individuals. ADL scores were not significantly different between groups (*p* = 0.06) (Table 1).

**Table 1:** Demographic characteristics and neuropsychological tests between NC and aMCI groups.

	NC(n = 26)	aMCI(n = 38)	*p* value
Age in years	64.6 ± 7.0	67.5 ± 7.2	0.115
Gender (Male/female)	11/15	15/23	0.693
Years of education	11.7 ± 2.3	11.1 ± 3.3	0.456
MMSE	28(27,29)	27(25,28)	0.001
MoCA	25(23,26)	21(19,23)	< 0.001
ADL	21(20,22)	21(20,23)	0.06
Memory			
AVLT immediate recall	25.0(22.0,30.3)	20.5(17.0,27.0)	0.003
AVLT delayed recall	10.0(8.8,11.0)	5.0(2.3,8.0)	< 0.001
AVLT recognition	13(12,14)	9(7,11)	< 0.001
ROCF immediate recall	21.5(18.0,25.3)	17.0(12.0,21.3)	0.003
ROCF delayed recall	20.8(17.5,24.3)	17.3(11.9,19.5)	0.002
Language			
BNT	27(24,28)	24(22,26)	0.001
VFT animal	20.0(17.8,22.3)	15.0(13.0,19.0)	< 0.001
Attention			
Forward digit span	8.0(6.8,9.0)	8.0(6.8,8.0)	0.262
Backward digit span	5(4,6)	4(3,5)	0.031
Visuospatial function			
ROCF copying	33.0(31.0,34.3)	32.0(29.4,33.3)	0.054
JLO (30 items)	24.5(23.4,26.8)	25.3(22.9,26.5)	0.902
Executive function			
TMT-A	36.0(29.8,44.5)	47.0(39.5,57.3)	0.002
TMT-B	60.0(46.3,79.3)	83.0(62.8,104.3)	0.002
Stroop word	80.5(69.0,88.0)	67.5(60.8,81.0)	0.021
Stroop color	63.0(55.0,70.0)	52.0(42.0,62.3)	0.001
Stroop word/color	31.0(27.8,34.3)	27.0(20.0,31.0)	0.003
GCF copying	17.0(16.0,17.3)	16.0(15.0,17.0)	0.134
GCF 3-min recall	16.0(15.0,16.6)	12.8(10.4,15.0)	< 0.001
GCF 20-min delayed recall	16(14,17)	13(10,15)	< 0.001

Values are expressed as mean ± standard deviation or median (interquartile range).

*n* number; *NC* Normal control individuals; *aMCI* Amnestic mild cognitive impairment; *MMSE* Mini-mental state examination; *MoCA* Montreal cognitive assessment; *ADL* Activity of daily living scale; *AVLT* Auditory verbal learning test; *ROCF* Rey-Osterrieth complex figure; *BNT* Boston naming test; *VFT* Verbal fluency test; *JLO* Judgement of line orientation (30 items in total); *TMT* Trail making test (part A or B, time in seconds); *GCF* Geriatric complex figure; Stroop word/color test, number of words completed in 45 s.

Compared with NC individuals, aMCI patients performed worse in memory, language, attention, and executive function, and the scores on these scales were significantly different between groups (all *p* < 0.05). NC individuals and aMCI patients did not significantly differ in visuospatial scores (ROCF copying:* p* = 0.054; JLO: *p* = 0.902) (Table 1).

### Pearson correlation between GCF and classic ROCF test total scores

**Figure 2:**
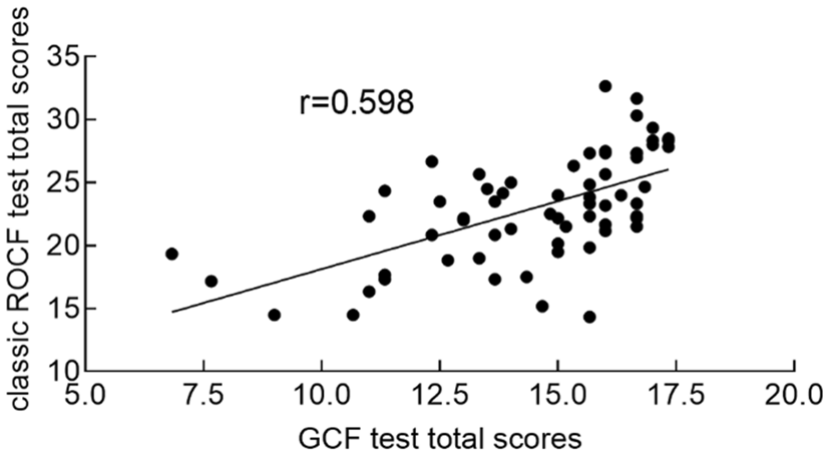
Pearson correlation between GCF and classic ROCF test total scores. All subjects completed both tests, including copying, 3-min recall, and 20-min delayed recall. We manually scored each GCF or ROCF test unit in terms of accuracy and location using Osterrich's scoring. There was a significant correlation between GCF and classical ROCF test total scores (r = 0.598;* p* < 0.001).

There was no significant difference between groups in GCF and classical ROCF copying test scores (all *p* > 0.05). In GCF (all* p* < 0.001) and ROCF (all *p* < 0.01) 3 min recall and 20 min delayed recall tests, the scores of aMCI patients were significantly lower than NC individuals (Table 1). Therefore, we took the average scores of copying, 3-min recall, and 20-min delayed recall as the total score of each test. The total score of the GCF test was significantly correlated with the classical ROCF test (r = 0.598, *p* < 0.001; Fig. 2), indicating that they were comparable in assessing the visuo-constructional ability and visual memory of the elderly.

### Performance characteristics of digital GCF test in patients with aMCI and NC individuals

**Figure 3:**
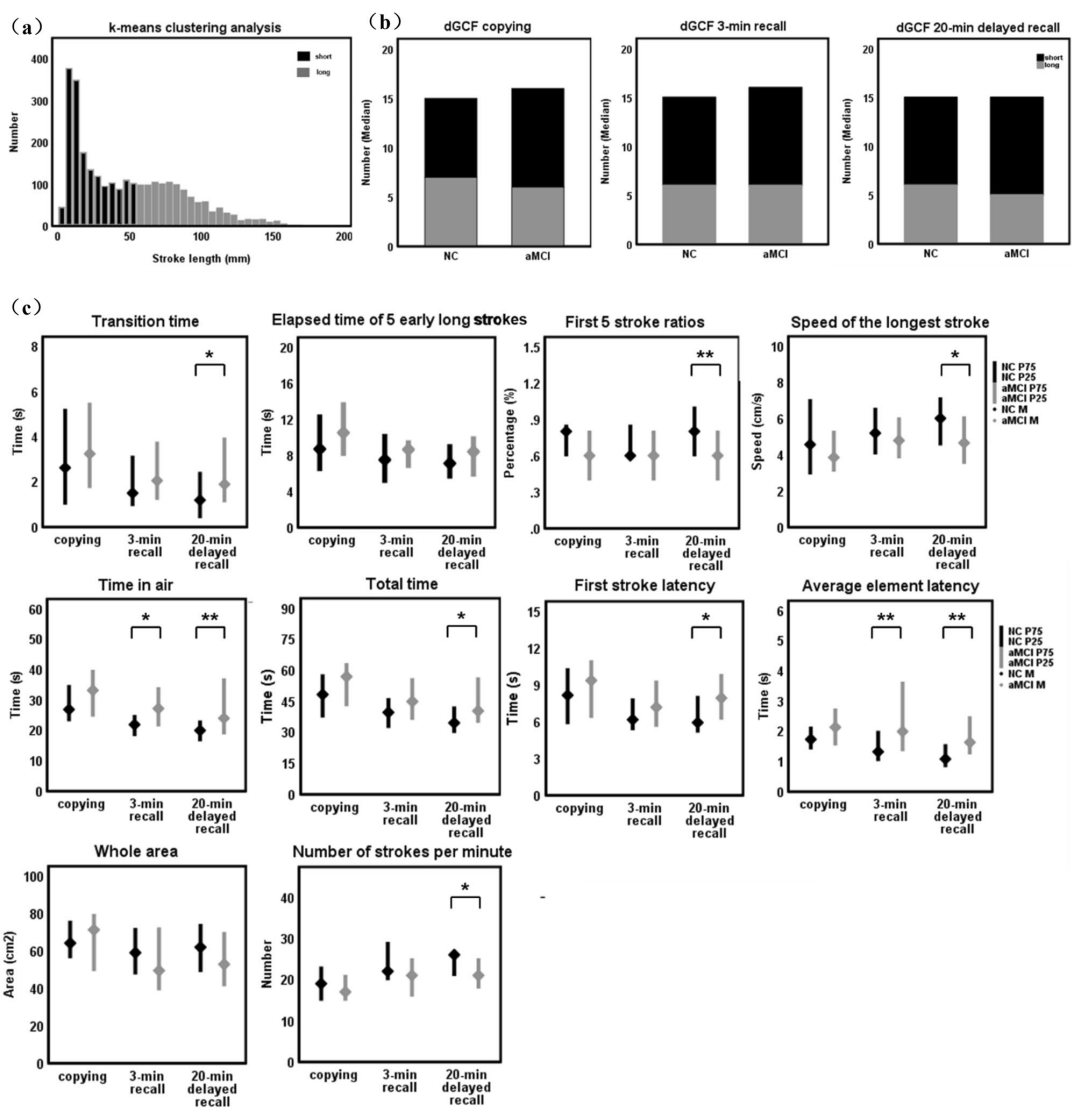
Pen stroke analysis module of dGCF software. Extract the number, length, speed, and interval time of strokes in the drawing process, and then obtain dGCF parameters through automatic or semi-automatic analysis. (**a**) short (black)/Long (grey) strokes were classified by k-means clustering with a cut-off value of 55.72 mm. (**b**) Difference analysis of long (grey), short (black) strokes, and total strokes between NC and aMCI groups in copying, 3-min recall, and 20-min delayed recall. (**c**) Lateral comparison and longitudinal trend analysis of dGCF parameters between NC (black) and aMCI (grey) groups.*p < 0.05, **p < 0.01. dGCF, digital Geriatric Complex Figure.

We included the total number of copying, 3-min recall, and 20-min delayed recall test lines for long/short stroke classification analysis using the k-means clustering method, with a cut-off value of 55.72 mm (Fig. 3a). In 20-min delayed recall, the number of short strokes of aMCI patients was significantly higher than NC individuals (*p* = 0.38; Fig. 3b). However, the number of long/short strokes differed significantly between groups in copying and 3-min recall (all *p* > 0.05; Fig. 3b).

Analysis of dGCF variables between the groups found no significant difference in the copying test (all *p* > 0.05). In 3-min recall, the time in air and average element latency of aMCI patients were significantly longer than that of NC individuals (all *p* < 0.05). And in 20-min delayed recall, we found a significant difference in transition time, first 5 stroke ratios, speed of the longest stroke, time in air, total time, first stroke latency, average element latency, and strokes per minute (all *p* < 0.05). The whole area and elapsed time of 5 early long strokes were not different between patients with aMCI and NC individuals (all *p* > 0.05) (Fig. 3c; Table 2).

**Table 2:** Comparison of dGCF parameters between aMCI patients and NC individuals in copying, 3-min recall, and 20-min delayed recall.

Digital parameters	NC(n = 26) copying/3-min recall/20-min delayed recall	aMCI(n = 38) copying/3-min recall/20-min delayed recall	*p *value
Transition time (s)	2.62(1.02,5.20)	3.24(1.75,5.47)	0.385
	1.50(0.95,3.13)	2.05(1.23,3.75)	0.187
	1.19(0.42,2.41)	1.89(1.12,3.93)	**0.043**
Elapsed time of 5 early long strokes (s)	8.69(6.39,12.37)	10.49(8.07,13.72)	0.199
	7.49(5.07,10.22)	8.63(6.73,9.51)	0.623
	7.10(5.56,9.09)	8.39(5.77,9.95)	0.305
First 5 stroke ratios (%)	0.8(0.6,0.85)	0.6(0.4,0.8)	0.122
	0.6(0.6,0.85)	0.6(0.4,0.8)	0.20
	0.8(0.6,1)	0.6(0.4,0.8)	**0.009**
Speed of the longest stroke (cm/s)	4.56(2.94,7.03)	3.85(3.10,5.28)	0.286
	5.19(4.05,6.55)	4.78(3.84,6.02)	0.712
	6.00(4.54,7.13)	4.65(3.53,6.07)	**0.039**
Average drawing speed (cm/s)	4.17(3.04,4.62)	3.66(3.12,4.40)	0.268
	4.11(3.50,5.35)	3.89(3.25,4.99)	0.412
	4.67(3.56,5.81)	3.95(3.37,4.59)	0.101
Time in air (s)	26.83(23.15,34.67)	33.14(24.69,39.65)	0.085
	21.85(18.26,24.81)	27.15(21.49,33.94)	**0.011**
	19.93(16.59,23.00)	23.94(18.86,36.87)	**0.006**
Total time (s)	48.22(37.47,57.72)	56.88(42.87,63.08)	0.107
	39.61(32.37,46.18)	44.87(36.30,55.79)	0.065
	34.52(29.96,42.15)	40.30(34.83,56.21)	**0.01**
First stroke latency (s)	8.15(5.85,10.3)	9.35(6.35,10.95)	0.28
	6.16(5.35,7.83)	7.17(5.62,9.29)	0.144
	5.92(5.16,8.04)	7.92(6.20,9.83)	**0.018**
Average element latency (s)	1.73(1.42,2.13)	2.13(1.55,2.73)	0.08
	1.32(1.03,1.99)	1.99(1.36,3.62)	**0.007**
	1.08(0.83,1.55)	1.63(1.25,2.47)	**0.002**
Whole area (cm^2^)	64.18(56.45,75.76)	71.27(49.63,79,43)	0.444
	59.03(47.77,71.89)	49.49(39.31,72.20)	0.234
	61.98(49.06,74.04)	52.86(41.44,69.73)	0.204
Strokes per minute (n)	19(15,23)	17(15,21)	0.32
	22(20,29)	21(16,25)	0.173
	26(21,27)	21(18,25)	**0.006**

Significant values are in bold.

Values are expressed as median (interquartile range).

*n* Number; *NC* Nomal control individuals; *aMCI* Amnestic mild cognitive impairment; *s* Second; *cm*^*2*^ Square centimeter; *dGCF* Digital geriatric complex figure.

In the longitudinal analysis, we found that with the extension of time, although the drawing performance of the two groups tended to improve, the aMCI patients still performed worse than the NC individuals after 20 min (Fig. 3c).

### Partial correlation analysis between dGCF parameters and cognitive domain composites

We compared the correlation between dGCF parameters and cognitive domains in 20-min delayed recall. After adjusting for age, sex, and education level, the transition time was negatively correlated with attention (*r* = − 0.292,* p* = 0.019) and executive function (*r* = − 0.275,* p* = 0.032). The speed of the longest stroke was positively correlated with executive function (*r* = 0.302, *p* = 0.015). The time in air was moderately negatively correlated with attention (*r* = − 0.408, *p* = 0.001) and executive function (*r* = − 0.448, *p* < 0.001). Also, the total time was negatively correlated with attention (*r* = − 0.342, *p* = 0.007) and executive function (*r* = − 0.429, *p* = 0.001). The first stroke latency was negatively correlated with memory (*r* = -0.365, *p* = 0.004) and visuospatial function (*r* = − 0.407, *p* = 0.001). The average element latency was moderately negatively correlated with memory (*r* = − 0.377, *p* = 0.003) and attention (*r* = − 0.33, *p* = 0.009). The number of strokes per minute was positively correlated with multiple cognitive domains (all *p* < 0.05) (Table 3).

**Table 3:** Partial correlation analysis between dGCF parameters and cognitive domain composites.

Cognitive domain (z-score)	Transition time *p*	First 5 stroke ratios *p*	Speed of the longest stroke *p*	Time in air *p*	Total time *p*	First stroke latency *p*	Average element latency *p*	Strokes per minute *p*
Memory	− 0.133	0.11	− 0.018	− 0.26	− 0.181	− 0.365	− 0.377	0.326
	0.307	0.397	0.891	**0.043**	0.163	**0.004**	**0.003**	**0.01**
Language	− 0.148	0.242	0.07	− 0.22	− 0.12	− 0.199	− 0.17	0.126
	0.254	0.06	0.59	0.089	0.358	0.115	0.191	0.335
Attention	− 0.292	0.095	0.081	− 0.408	− 0.342	− 0.183	− 0.33	0.311
	**0.019**	0.469	0.532	**0.001**	**0.007**	0.159	**0.009**	**0.015**
Executive function	− 0.275	0.217	0.302	− 0.448	− 0.429	− 0.23	− 0.217	0.376
	**0.032**	0.093	**0.015**	** < 0.001**	**0.001**	0.074	0.093	**0.003**
Visuospatial function	− 0.226	0.17	0.133	− 0.264	− 0.273	− 0.407	− 0.166	0.263
	0.08	0.189	0.307	0.04	0.033	**0.001**	0.202	**0.04**

Significant values are in bold.

z-score, this study selected neuropsychological tests of interest in each cognitive domain. All tests were converted to standard z-scores using published criteria, and then composite scores for each cognitive domain were created by averaging each category.

### Diagnostic value of dGCF parameters and cGCF scores in discriminating patients with aMCI from NC individuals

In the 20-min delayed recall test, we selected the transition time, first 5 stroke ratios, speed of the longest stroke, time in air, total time, first stroke latency, average element latency, and strokes per minute into the positive stepwise logistic regression model. The results showed that the significant odds ratios (95% confidence interval) of the average element latency (3.776[1.322–10.785]) and first 5 stroke ratios (0.038[0.003–0.559]) were the best predictor variable (all *p* < 0.05; Table 4), and other variables were excluded.

**Table 4:** Logistic regression models of dGCF parameters and cGCF scores for discriminate patients with aMCI from NC individuals.

Model	Selected variable	Beta (β)	Sβ	SE	WaldX^2^	OR (95%CI)	*p *value
a. dGCF	Average element latency	1.329	0.494	0.535	6.156	3.776 (1.322–10.785)	0.013
	First 5 stroke ratios	− 3.27	− 0.287	1.372	5.682	0.038 (0.003–0.559)	0.017
b. cGCF	cGCF score	− 0.408	− 0.424	0.128	10.163	0.665(0.518–0.855)	0.001
c. (d + c) GCF	Average element latency	1.396	0.832	0.602	5.374	4.037 (1.241–13.138)	0.022
	First 5 stroke ratios	− 3.274	− 0.460	1.72	3.623	0.038 (0.001–1.00)	0.021
	cGCF score	− 0.392	− 0.691	0.145	7.294	0.676 (0.508–0.898)	0.007

*NC* Normal control individuals; *aMCI* Amnestic mild cognitive impairment; *dGCF* Digital geriatric complex figure; *cGCF* Conventional geriatric complex figure; *β* Logistic regression coefficient; *Sβ* standard logistic regression coefficient; *SE* Standard error; *OR* Odds ratio; *CI* Confidence interval of to odds ratio.

**Figure 4:**
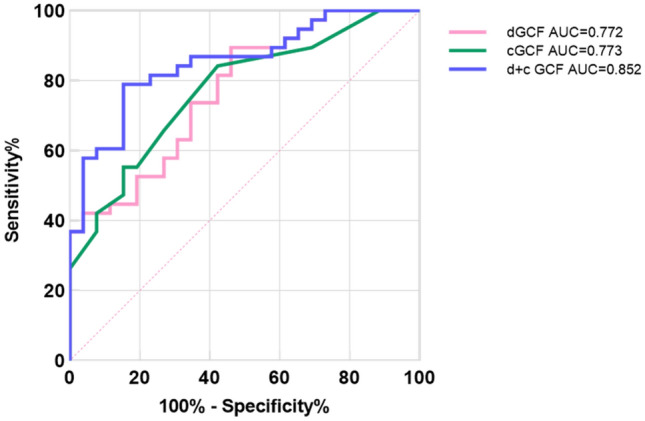
AUCs for aMCI patients against NCs using dGCF model (average element latency and first 5 stroke ratios), cGCF score, or (d + c) model.

Predicting patients with aMCI and NC individuals, the combination of average element latency and first 5 stroke ratios (Table 4, model a) correctly classified 70.3% of cases (AUC:0.772; *p* < 0.001; Table 5; Fig. 4), with a sensitivity of 89.47% and a specificity of 53.85%, and an optimal cut-off of 0.43. when cGCF score (Table 4, model b) was used to discriminate between aMCI patients and NC individuals at a cut-off value of 15.5, showing a sensitivity of 84.21%, a specificity of 57.69%, and an accuracy of 73.4%(AUC:0.773; *p* < 0.001; Table 5; Fig. 4). Finally, we found that the combination of average element latency, first 5 stroke ratios, and cGCF score (Table 4, model c) can significantly improve the specificity of diagnosis. 78.1% (AUC:0.852; *p* < 0.0001; Table 5; Fig. 4) of aMCI patients and NC individuals were correctly classified, with a sensitivity of 78.95% and a specificity of 84.62%. The best cut-off value is 0.59.

**Table 5:** Diagnostic value of classification models in discriminating patients with aMCI from NC individuals.

Model	ROC AUC	Sensitivity	Specificity	Accuracy	*p *value
a. dGCF	0.772	89.47	53.85	70.3	< 0.001
b. cGCF	0.773	84.21	57.69	73.4	< 0.001
c. (d + c) GCF	0.852	78.95	84.62	78.1	< 0.0001

*ROC* Receiver operating characteristics curve; *AUC* Area under the cure.

## Discussion

This study uses a digital pen and tablet to extract the multi-dimensional kinematic parameters involved in the drawing process, aiming to clarify the organization strategy of drawing complex figures for aMCI patients and establish a rapid, objective, and sensitive digital scoring system that could replace traditional scoring. First, we compared the GCF test total score with the classical ROCF to verify the validity of the GCF data in the Chinese population. Subsequently, we performed a horizontal comparison and longitudinal analysis of dGCF parameters in aMCI patients and NC individuals. The final aim of the study was to evaluate the potential of dGCF variables in differentiating aMCI patients from NC individuals compared to cGCF scores.

A previous study reported that GCF test scores were significantly correlated with RBANS' complex figure copying and delayed recall scores^19^. And its copying, recall, and strategy scores are well-distributed among healthy older people (over 60 years old). In line with this study, the GCF test total score is strongly correlated with the classical ROCF test, indicating that they are comparable in assessing the visuo-constructional ability and visual memory of the elderly. Other studies have shown that the simplified figures can also detect significant heterogeneity in patients of MCI, AD, and NC individuals^20, 45–47^. In addition, a study found significant outliers in the ROCF copy scores of AD patients, which may be related to decreased enthusiasm and coordination when encountering a complex figure due to severe visuospatial impairment^20^. These findings mean that the simplified figure may have a better application prospect for the low education level, the elderly, and the digital transformation of the scale.

We found that the 3-min and 20-min delayed recall scores of ROCF and GCF tests in aMCI patients were significantly lower than those of NC individuals, so we further analyzed the digital variables of 3-min and 20-min recall between the two groups.

The results showed that the time in air and average element latency of aMCI patients were significantly longer than that of NC individuals in 3-min recall. In 20-min recall, the transition time, time in air, total time, first stroke latency, and average element latency of aMCI patients were significantly longer than those of NC individuals. The first 5 stroke ratios, speed of the longest stroke, and strokes per minute were lower than those of NC individuals. And aMCI patients used more short strokes in drawing than NC individuals. It suggests that poor drawing performance of aMCI patients may be related to abnormal organizational strategies. If the entire process of the drawing task is digitally captured, it may reveal subtle signs of cognitive impairment.

GCF consists of the main frame and five local elements. Wilson and Batchelor^48^ found that more than 50% of NC individuals draw the main frame first and add local elements later. Drawing in a fragmented and chaotic way may suggest damage to the prefrontal cortex, reflecting that individuals cannot effectively integrate global information. The drawing of the main frame requires at least 5 strokes (rectangle, horizontal/vertical/diagonal lines). Since the longest stroke is likely to be used to build a graphic frame, the slowness of drawing the longest stroke may imply impaired executive function and visuo-constructional ability. The increase in the transition time or the decrease in the first 5 stroke ratio also suggests that patients with aMCI begin to draw local features or details earlier, which can also reflect the impairment of executive function and attention. In the early AD stage, memory retrieval impairment leads to the decline of motor execution and motor integration, called executive dysfunction syndrome^49^. Some studies also tend to attribute the preferential extraction of local features in AD patients to mild simulated agnosia, which is part of Balint syndrome^50^. In addition, we found that patients with aMCI were hesitant at the beginning of drawing or adding local elements and even missed some critical structures. Therefore, the increase in the first stroke latency and average element latency may indicate the impairment of visuospatial working memory and attention. All time variables reflect the intermittence in the process of drawing or execution. Especially after 20 min, it needs to be converted into motor skills and execution plans through visuospatial working memory^51^. Our cognitive domain analysis of digital variables also suggests that the first stroke latency and average element latency are significantly related to memory and attention. It may be related to the disorders of the frontal lobe and temporal-parietal lobe brain regions, which will affect the visuospatial working memory, decision-making, and cognitive flexibility, and then interfere with the accuracy of drawing results^24, 52, 53^. These findings suggest that the poor drawing performance in 3-min recall in aMCI patients may be related to impaired visuospatial memory, and the drawing strategy is acceptable. However, at an interval of 20 min, strategies for constructing visual components of aMCI patients were also significantly affected, resulting in the inaccuracy of the finished product map.

Using the combination model of the first 5 stroke ratios and average element latency can better distinguish aMCI patients and NC individuals (accuracy: 70.3%), which is equivalent to the time-consuming cGCF score (accuracy: 73.4%), and its sensitivity is slightly higher than the cGCF score (dGCF: 89.47%, cGCF: 84.21%). Therefore, we constructed the regression equation of the above model: **ln** (***p*/**(**1-*p***)) = 0.536 + 1.329 * x_**1**_-3.27 * x_**2**_ (***p***: probability of illness, **x**_**1**_: average element latency, **x**_**2**_: first 5 stroke ratios), in which **x**_**1**_ is the most critical variable (standardized beta: **x**_**1**_ = 0.494, **x**_**2**_ = − 0.287). It can be found that the variables reflecting visuospatial working memory and visual component construction strategies show the best model fitting. Moreover, the combination model of the dGCF variables and cGCF score can significantly improve the accuracy and specificity of distinguishing aMCI patients from NC individuals (accuracy: 78.1%, specificity: 84.62%). The regression equation of this model is **ln** (***p*/**(**1-*p***)) = 6.521 + 1.396 * x_**1**_-3.724 * x_**2**_-0.392 * x_**3**_ (***p***: probability, **x**_**1**_: average element latency, **x**_**2**_: first 5 stroke ratios, **x**_**3**_: cGCF score), in which **x**_**1**_ is the most critical variable (standardized beta: **x**_**1**_ = 0.832, **x**_**2**_ = -0.46, **x**_**3**_ = -0.691). The above two regression equations can be used to identify patients with MCI in clinical application. Although the efficiency of the pure digital variable model to identify patients with aMCI is slightly low, its application prospect is still worth looking forward to. We will develop more digital variables related to visuospatial ability in the future, and multivariate combinations may optimize the model.

In previous digital clock drawing test (dCDT) studies, Yuan et al. ^54^ found that acquired dCDT features correlated with brain volume. Moreover, the combined model of clinical risk factors, dCDT composite scores, and MRI measures can distinguish the patients with MCI well from the normal cognitive individuals, and the AUC is 0.897. Another study from Harvard University's Aging Brain Institute found among normal participants with biomarkers, the dCDT summary score was associated with more significant amyloid and tau burden and showed better discrimination between Aβ ± groups than the Primary Alzheimer Cognitive Composite^55^. Therefore, digital measurement may be an effective tool for detecting early cognitive changes in AD trajectories. GCF is similar to a clock. We will further validate the potential of dGCF as a biomarker for patients with MCI by combining neuroimaging, body fluid, and PET-CT biomarkers.

This study also has some potential limitations. First, the relatively small sample size of subjects may lead to the selection bias of digital variables and slightly reduce the efficiency of the diagnostic model. To overcome this difficulty, all subjects recruited in this study received brain imaging and a standardized neuropsychological battery and were rigorously diagnosed by professional neurologists. Second, as a newly developed cognitive assessment tool, it has not been evaluated for reliability and validity in large populations, such as retesting and internal consistency. However, similar studies have been published in recent years. We also plan to use this tool in community screening in China to verify its reliability, validity, and clinical applicability. Finally, the standardized neuropsychological battery took a long time, which decreased the subjects' enthusiasm and cooperation in the test. Especially in the 20-min recall test, they showed impatience or slightly refused, affecting the final drawing results. However, we still found that the simplified figure is more accessible to implement than the ROCF.

In conclusion, dGCF can conduct a multi-dimensional evaluation after digitizing the scale by extracting dynamic parameters such as the number, length, speed, and time of strokes to better analyze the subjects' drawing behavior patterns. As a supplement to traditional scoring, digital puls conventional can significantly improve the discrimination of aMCI patients.

We believe that this highly effective cognitive screening tool can be used to identify people at high risk of dementia (supplementary information).

### Supplementary Information

Supplementary Information.

## Data Availability

The datasets generated during and analyzed during the current study are available from the corresponding author upon reasonable request.
