# Supplementary material for: A tablet-based multi-dimensional drawing system can effectively distinguish patients with amnestic MCI from healthy individuals
Source: Sci Rep. 2024 Jan 10;14:982. doi: 10.1038/s41598-023-46710-y (PMC10781783; doi:10.1038/s41598-023-46710-y)
Supplement: Supplementary file 1 — Supplementary Information. [file 41598_2023_46710_MOESM1_ESM.pdf]

A Tablet-based Multi-dimensional Drawing System Can Effectively Distinguish Patients with Amnestic MCI from Healthy Individuals

Xiaonan Zhang[2, 3] Liangliang Lv[5] Jiani Shen[4] Jinyu Chen[4] Hui Zhang[2, 3, 6*] Yang Li[1*]

[1]Department of Neurology, First Hospital of Shanxi Medical University, Taiyuan, China

[2]Department of Radiology, First Hospital of Shanxi Medical University, Taiyuan, China

[3]Department of Medical Imaging, Shanxi Medical University, Taiyuan, China

[4]Department of First Clinical Medicine, Shanxi Medical University, Taiyuan, China

[5]Lvliang People's Hospital, Lvliang, China

[6]Shanxi Key Laboratory of Intelligent Imaging and Nanomedicine, First Hospital of Shanxi Medical University, Taiyuan, China

* Correspondence: Yang Li, 15035182003@163.com; Hui Zhang, zhanghui_mr@163.com; These two authors contributed equally to this work and share corresponding authorship.

Date	Group(0=nomal, 1=aMCI)	Sex(1=Female, 2=male)	Age in years	Years of education	MMSE(score)	MoCA (score)	CDR(score)	ADL(score)	Memory :	AVLT immediate recall(number)	AVLT 20-min delayed recall(number)	AVLT recognition(number)	ROCF immediate recall(score)	ROCF delayed recall(score)
2021-01-25	1	1	54	16	25	18	0.5	23		10	0	11	17	16.5
2021-01-27	1	1	79	6	24	16	0.5	24		18	6	9	8	12.5
2021-02-05	1	1	75	14	25	26	0.5	21		35	5	9	18.5	16.5
2021-02-05	1	2	84	17	25	24	0.5	21		19	4	7	20	15.5
2021-02-23	1	2	77	12	27	22	0.5	20		12	0	5	21	19
2021-02-23	1	1	74	9	25	22	0.5	22		21	4	8	19	18
2021-02-27	1	1	68	12	26	24	0.5	22		28	8	9	10	9
2021-03-05	0	1	64	9	29	26	0	20		22	9	14	18	17.5
2021-03-13	0	1	74	13	30	26	0	21		29	13	12	18	18
2021-03-13	1	1	63	12	29	25	0.5	20		28	10	11	25.5	22.5
2021-03-13	1	2	63	9	27	21	0.5	25		11	5	14	12	11.5
2021-03-17	1	1	68	15	30	23	0.5	20		27	7	9	16	14.5
2021-03-21	1	2	72	12	28	21	0.5	20		19	0	5	9.5	9
2021-03-23	1	2	72	9	24	21	0.5	21		21	0	11	18	17
2021-03-25	1	2	65	9	26	20	0.5	22		20	4	7	10.5	8.5
2021-03-27	0	2	69	9	27	23	0	20		16	6	10	22	19
2021-03-27	1	1	68	6	26	21	0.5	21		15	4	9	13	10.5
2021-03-29	0	1	64	12	27	24	0	21		25	10	11	21	20.5
2021-03-31	1	2	65	15	29	20	0.5	23		31	7	8	17	19.5
2021-04-02	0	1	60	15	27	25	0	20		25	11	13	25	23.5
2021-04-07	0	1	62	9	29	25	0	22		35	13	14	16.5	21.5
2021-04-09	1	1	66	9	27	18	0.5	20		28	8	11	22.5	20.5
2021-04-09	1	1	60	7	26	18	0.5	20		17	11	6	16	18.5
2021-04-11	0	2	63	12	27	28	0	21		21	8	12	33	32
2021-04-11	1	2	60	9	25	18	0.5	23		19	0	5	20	19.5
2021-04-11	1	2	60	9	28	22	0.5	20		19	3	11	25.5	21.5
2021-04-13	1	1	71	6	25	17	0.5	21		15	0	6	5.5	10.5
2021-04-16	0	1	55	12	28	24	0	20		17	10	12	15	12.5
2021-04-17	0	2	70	12	29	26	0	21		27	10	14	25	24
2021-04-17	1	1	70	7	25	22	0.5	21		27	7	13	19.5	19.5
2021-04-19	0	1	66	10	30	28	0	23		25	8	13	25	26
2021-04-19	0	2	74	16	29	26	0	20		22	6	12	24	26

2021-04-26	0	1	68	13	29	25	0	22		30	9	13	20.5	19.5
2021-04-26	0	1	82	13	29	26	0	24		32	11	14	13	13.5
2021-05-01	0	1	58	15	28	28	0	20		22	10	13	18.5	16.5
2021-05-10	1	1	77	16	27	25	0.5	20		26	6	13	25	23
2021-05-10	1	2	82	15	27	24	0.5	27		21	5	6	23	23
2021-05-11	1	1	57	14	24	21	0.5	27		15	0	10	21	18.5
2021-05-15	0	2	69	9	28	26	0	20		25	5	11	27	25
2021-05-15	0	1	54	11	25	22	0	20		22	10	10	19.5	18.5
2021-05-15	0	2	51	11	28	23	0	20		31	11	12	26	27
2021-05-19	1	1	75	8	25	18	0.5	34		15	4	10	11.5	10.5
2021-06-03	0	1	72	12	27	27	0	22		34	13	14	23	21
2021-06-04	0	1	62	12	30	24	0	21		30	12	14	15.5	10
2021-06-05	1	2	65	12	25	22	0.5	22		24	0	12	16.5	17
2021-06-10	1	1	61	9	28	23	0.5	22		19	8	9	24	25
2021-06-15	1	1	64	13	30	20	0.5	21		25	5	10	14.5	18
2021-06-22	1	1	66	15	27	24	0.5	23		27	11	9	12	12
2021-06-24	1	2	73	12	27	19	0.5	21		22	0	3	9	8
2021-06-30	0	1	67	14	27	21	0	23		27	7	10	26.5	22
2021-07-07	0	2	63	12	29	23	0	21		27	10	12	30	30
2021-07-12	0	1	59	15	28	26	0	20		32	11	14	28.5	23
2021-07-13	1	1	66	16	26	16	0.5	20		26	3	2	16	15
2021-07-14	1	2	68	16	28	20	0.5	22		14	6	13	26	22
2021-07-15	0	2	61	12	30	25	0	20		21	9	14	23	24
2021-07-15	1	1	64	9	27	13	0.5	21		17	6	8	15	13
2021-07-20	1	1	72	9	24	21	0.5	23		23	8	13	16	17.5
2021-07-21	0	1	58	7	26	22	0	21		35	14	14	18	17
2021-07-26	1	2	57	9	30	20	0.5	21		20	6	8	12	11
2021-08-05	0	2	65	9	29	21	0	22		25	9	13	21	20.5
2021-09-25	1	1	58	9	30	24	0.5	21		32	13	13	28	28
2021-10-15	0	1	70	9	28	23	0	20		25	11	12	16	17.5
2021-10-16	1	2	64	9	27	22	0.5	22		19	8	11	17.5	17.5
2021-10-18	1	1	62	12	28	22	0.5	20		29	10	13	22	19

Language:	BNT(score)	VFT animal(number)	Attention:	Forward digit span(number)	Backward digit span(number)	Executive function:	TMT-A (s)	TMT-B(s)	Stroop word(number)	Stroop color(number)	Stroop word/color(number)	Visuospatial function:	ROCF copying(score)
	24	14		8	4		33	88	65	39	29		29
	17	14		7	3		105	84	46	40	29		32
	22	22		7	6		89	102	74	55	27		32
	25	13		9	5		63	86	53	43	15		34
	22	19		8	6		96	145	63	32	20		31.5
	20	15		8	4		57	76	66	46	17		30
	26	23		8	4		37	39	89	72	38		33
	19	18		5	4		46	67	70	55	29		35
	26	17		6	5		78	79	78	63	33		30
	23	14		7	3		56	98	90	66	38		34
	26	24		7	3		47	70	79	52	27		19.5
	24	18		8	5		27	36	98	75	37		33
	18	11		9	5		54	83	72	69	15		33
	23	16		6	3		47	120	57	37	18		29
	27	17		8	6		40	58	81	63	40		24.5
	25	21		6	4		35	80	60	45	24		31
	24	16		8	3		47	50	71	56	28		33
	27	17		8	2		35	89	64	53	30		29
	24	21		6	3		24	36	83	51	20		30
	27	20		8	6		32	52	81	68	40		34
	27	18		8	5		54	51	70	80	32		32
	24	19		9	3		58	75	52	47	21		32
	24	15		9	6		47	193	69	55	27		32
	28	28		9	5		43	60	87	66	25		33
	24	13		5	4		51	120	42	35	25		33
	23	18		8	4		40	62	81	46	12		30
	22	12		5	3		86	170	41	42	23		33
	24	26		8	3		29	48	53	57	27		31
	29	22		9	6		25	86	85	77	29		30
	25	17		8	3		49	91	78	54	26		33
	28	21		10	5		28	39	93	73	45		31
	28	25		10	8		41	61	93	54	33		35

	24	17		7	5		36	61	87	65	30		34
	28	20		9	8		36	40	80	55	31		33
	28	23		8	8		37	41	100	81	50		30
	26	23		8	5		70	104	67	54	22		34
	28	11		9	4		50	63	53	41	14		34
	19	15		5	4		45	105	67	45	9		34
	23	18		8	3		25	38	80	58	26		33
	27	25		7	4		47	54	65	60	31		32
	27	25		9	5		30	44	92	76	51		35
	23	10		7	3		69	148	43	47	28		21.5
	27	19		6	5		64	70	63	62	31		33
	23	20		7	3		51	56	76	63	26		35
	25	13		6	3		41	62	64	54	31		29
	27	13		8	3		54	69	100	78	41		35
	26	19		9	5		32	44	100	79	32		32
	26	20		7	6		40	108	92	84	21		33
	22	16		8	6		38	76	97	78	31		35
	26	17		9	5		25	47	84	66	34		35
	28	20		7	6		35	65	66	55	20		35
	28	18		9	8		36	41	85	73	33		34
	19	12		8	5		31	70	64	42	14		27
	25	9		8	3		54	96	74	60	38		34
	25	21		10	5		44	60	91	53	38		34
	16	13		5	4		59	165	54	41	29		25
	27	15		8	4		45	83	67	57	27		33
	20	17		3	1		44	83	74	63	35		32
	18	12		3	4		56	91	64	51	24		22.5
	28	12		6	4		26	83	83	62	28		33
	26	17		8	3		46	64	68	62	41		35
	19	18		8	4		37	115	91	69	29		34
	26	19		6	3		34	45	71	52	27		29.5
	20	14		7	3		30	63	62	38	21		32

JLO (30 items)(score)		GCF copying (score)	GCF 3-min recall(score)	GCF 20-min delayed recall(score)		dGCF copying:	Transition time (ms)	Elapsed time of 5 early long strokes (ms)	First 5 stroke ratios (%)	Time in air (ms)	Total time (ms)	First stroke latency (ms)	total strokes (n)	Speed of the longest stroke (mm/s)
27		18	16	14			2523	6293	1	38010	57671	6220	14	90.1
22.5		18	16	16			5592	8752	0.8	19272	35902	2939	14	48.74
24.5		17	16	15			5026	19283	0.6	26647	51737	12584	11	22.81
22.5		17	16	16			0	3512	1	23197	34056	8221	15	119.5
26.5		17	15	14			11805	10916	0.6	30986	51151	6805	16	30.39
23.5		17	16.5	17			1588	5660	0.6	23594	36645	5881	17	79.46
25		18	17	17			1122	8313	0.8	27009	48758	8523	15	31.92
22		18	16	17			7290	13862	1	35995	61225	8509	15	26.58
24		14	11.5	12			3203	8618	0.6	26114	39764	12438	14	32.95
25		16	16	15			3175	22069	0.8	29973	64589	9349	14	24.02
24		18	16	16			2128	9777	0.4	32649	57855	7433	17	41.9
26.5		15	17	18			18895	8136	0	77317	101528	14901	19	42.42
27		15	16	16			5273	8434	0.6	23893	40903	5550	16	64.8
27		16	15	13.5			2757	11176	0.8	23114	42436	8654	14	42.1
29		15	12	12			0	15287	1	34553	64419	10052	14	23.02
24.5		17	15	16			20174	17774	0.6	52112	85819	17568	13	16.08
27.5		18	18	11			5176	8164	0.8	26568	45755	6517	15	51.01
23.5		17	17	18			715	6425	0.8	42565	57090	11033	15	65.11
26.5		16	14	11			0	9894	1	28163	47102	8077	14	41.98
24.5		15	15	15			2612	19104	0.4	22100	50229	2519	14	15.08
23		16	16	16			2628	5849	0.6	20235	32512	7877	12	70.94
26		17	18	17			672	2159	0.6	14042	20908	5130	15	130.25
19.5		16	14	15			2073	10121	0.8	17427	34763	5740	15	58.13
25		17	16	17			1705	6914	1	23157	37742	8654	16	73.31
26		17	16	18			512	2469	0.4	28809	47680	4765	18	70.13
25.5		17	17	16			3584	11875	0.8	35028	53993	11457	14	50.56
21		16	12	12			4224	10524	0.8	45058	62452	6453	14	36.41
21		15	11	15			5665	13690	0.8	31502	52407	8031	15	38.93
26		17	16	17			7932	12652	0.8	44046	68968	10718	16	27.61
25.5		18	16	17			1989	10454	0.8	38751	62573	3553	16	42.77
29		17	17	16			4961	10887	1	67868	96729	24632	25	43.13
30		17	16	15			4945	5656	0	57114	84872	9420	22	21.69

25.5		16	17	14			1927	6553	0.6	31460	50372	7520	20	106.82
26.5		13	6	4			3227	9978	0.2	40181	61030	19287	16	29
27.5		18	9	7			1404	7206	0.6	17013	34777	5385	16	71.98
21.5		15	12.5	11.5			810	5863	0.8	16503	27187	4309	15	66.51
26.5		18	12.5	11			5884	13691	0.4	36269	59735	10314	17	18.59
28.5		17	12	11			1988	12220	0.8	13368	31365	3921	13	35.7
29		13	10	10			1481	7449	0.4	28890	44221	9921	14	51.72
24.5		15	15	12			2487	11217	0.8	33891	59127	10814	13	53.84
29		18	17	15			3762	12997	0.6	20780	40406	7174	13	31.67
22		17	16	15			4571	15652	0.6	25083	47374	9893	13	36.57
24.5		16	10.5	5.5			3335	10315	0.4	36213	52599	12266	16	37.82
17.5		15	12	11			1220	4724	0.8	24427	34162	4371	13	121.48
24		16	14	17			6869	15935	0.6	79384	112203	38019	19	29.04
24.5		15	13	13			7939	9831	0.6	41891	63902	11358	16	56.84
22.5		15.5	15	15			936	14852	0.8	34357	62433	13104	17	30.7
25.5		17	15	15			3131	16379	1	33791	69878	9122	17	27.57
19		17	15	16			1581	8241	0.2	27909	52749	8253	15	23.5
26		18	10	9			2521	12956	0.8	32498	58409	8677	17	38.29
29.5		18	10	9			0	9771	1	23985	42954	4426	14	43.24
24		17	12	11.5			2338	14435	0.8	29628	55004	10535	13	31.12
26		17	12	13			12342	20478	0.6	37776	66645	8552	12	40.91
25		11	11	11			5407	10924	0.4	36094	57154	9658	14	41.49
24.5		16	15	14			1135	9637	0.8	24780	44119	12207	13	31.24
22.5		16	17	17			1084	7123	0.6	21026	36555	7940	15	63.37
25.5		16	14	13			2941	7576	0.6	37955	56604	6050	20	46.31
19.5		16	5.5	5.5			4371	13806	0.6	39477	62807	12624	15	32.42
23		17	14	14			13927	17227	0.6	48266	85465	9953	20	37.13
20		14	4	2.5			1805	9529	0.4	22259	40387	9669	14	52.4
27		17	15	15			6308	9257	0.4	16201	31691	2349	14	38.61
24		14	10	10			13275	8574	0.6	42722	64355	15367	18	57.55
26.5		15	15	14			3981	15073	0.6	31577	58420	5722	15	23.31
23.5		14	10	10			3260	7255	0.4	28696	42613	9271	16	55.23

Strokes per minute (n)	Average drawing speed (mm/s)	Whole area (mm2)	long strokes(n)	short strokes(n)	Average element latency (ms)		dGCF 3-recall:	Transition time (ms)	Elapsed time of 5 early long strokes (ms)	First 5 stroke ratios (%)	Time in air (ms)	Total time (ms)	First stroke latency (ms)	total strokes (n)
15	47.7	9211.67	8	6	4239.4			389	6805	1	28867	45978	5509	15
23	45.45	6390.7	7	7	1225.4			1616	5794	0.6	11961	23338	4490	13
13	33.98	8686.24	6	5	901.6			0	10308	1	17347	33018	5130	13
26	76.73	8066.79	8	7	1804.6			1240	3068	1	21217	31546	6634	16
19	40.05	5809.22	8	8	1548.2			1044	9647	0.8	24790	39756	13916	13
28	55.35	5886.68	5	12	1865.2			1250	7235	0.6	18010	31706	5769	16
18	33.98	6064.07	7	8	1658.2			697	10195	1	19930	40369	5972	14
15	33.3	7520.6	8	7	2259.8			3031	4334	0.8	25045	39434	7551	14
21	45.23	6445.98	5	9	1726.2			1251	5205	0.6	53616	65987	14951	15
13	24.07	7315.52	7	7	1979.6			3511	13852	0.6	21342	48526	9596	15
18	30.38	5608.24	6	11	2100.6			3404	8242	0.4	24888	45207	5220	15
11	29.48	5656.84	4	15	2235			2315	9679	0.6	23452	42491	7306	17
23	42.3	5848.86	6	10	1834.11			2124	7558	0.6	33721	51452	8466	18
20	40.05	6810.68	7	7	1500.2			2134	7424	0.8	18345	32594	5600	16
13	27.68	6675.62	7	7	1689			0	14002	1	21624	44046	9308	12
9	21.38	5944.59	7	6	1552.2			3043	13368	0.2	33006	61618	8217	15
20	42.75	7567.43	7	8	1488.2			3738	6933	0.6	23497	39460	6473	14
16	45.23	4782.34	5	10	3104.4			1375	4669	0.2	21493	34602	5655	17
18	41.18	7601.65	7	7	2353.8			4754	9399	0.8	22353	39362	4559	14
17	23.4	5472.97	6	8	1958.2			3478	12144	0.4	22857	49132	3730	15
22	47.93	3758.91	6	6	1047.6			978	3009	0.6	16691	25672	7443	15
43	85.05	4480.41	4	11	1096.6			866	4093	0.8	16448	25833	5141	14
26	45.68	7251.63	7	8	744.8			2878	13589	0.6	22067	46769	5461	15
25	62.77	9802.01	8	8	1196			1128	5843	1	17412	30649	5387	15
23	30.38	3379.57	2	16	1729.6			563	1553	0.2	20770	35027	6340	18
16	44.1	8164.8	7	7	2937.4			3798	8208	0.8	24346	41147	7697	15
13	42.08	6137.88	6	8	5011.4			8150	7482	0.2	50556	67618	6964	19
17	39.83	7956.02	7	8	2115.6			3174	7304	0.2	55367	74721	6776	18
14	32.85	7517.81	5	11	1637.8			4027	8703	0.6	28222	45339	7863	16
15	36.45	7598.61	6	10	1953.2			2781	7186	0.8	27336	45027	6752	16
16	35.33	8645.33	8	17	2141.2			4450	13549	0.8	54095	81012	12125	17
16	20.93	3926.58	2	20	3653.6			1040	5467	0	26955	50696	5450	20

24	50.4	7766.18	6	14	2558.4			5562	9729	0.6	24315	44704	4153	19
16	30.82	4047.22	5	11	2872.6			29	4040	1	29594	41430	13331	20
28	38.7	4631.48	5	11	1161.6			1411	2247	0.2	32156	43950	3413	14
33	78.98	7986.45	7	8	1495.8			751	6981	0.6	21111	31466	4516	15
17	24.53	3291.89	4	13	1967.4			5583	9201	0.4	23244	37893	7322	16
25	41.4	7930.91	6	7	640.2			3320	11317	0.8	19996	37586	5107	14
19	36	4062.05	5	9	2003.333			1201	8581	0.8	17211	30029	6730	10
13	32.63	7004.37	8	5	2544			3180	8369	0.6	18550	36085	6734	15
19	43.88	8297.94	7	6	1133.4			1375	9088	0.8	25102	41046	7664	13
16	38.93	7898.82	6	7	1157.4			1994	11972	0.6	26102	45680	8524	12
18	50.63	7025.13	6	10	3153.75			693	8917	0.8	23563	36401	8276	12
23	67.05	4039.88	5	8	2837.25			1612	4789	0.8	27936	35962	6570	10
10	35.77	12263.91	9	10	2930.6			6183	9438	0.6	30572	50300	8932	16
15	36.68	8981.28	8	8	2721.6			2971	8842	0.8	26347	43899	9265	16
16	31.73	10041.98	7	10	2685.6			2689	12738	0.8	46416	68714	20283	17
15	27.45	7679.81	8	9	2304.2			1224	12597	0.8	22457	51229	6908	17
17	20.25	2927.54	3	12	1939.2			3664	7117	0.4	28440	49949	7020	15
17	26.55	4975.02	6	11	2312.6			1440	8069	0.6	12624	26355	4205	11
20	37.13	5401.89	6	8	2145.71			2888	9393	0.6	46006	64550	5679	14
14	31.28	6020.58	7	6	1376.8			752	12106	0.8	65020	86585	16060	16
11	29.7	8262.61	6	6	2962.2			4750	10880	1	30797	49976	3977	10
15	38.93	6930.51	6	8	3318.333			558	5963	0.6	22425	40882	9360	16
18	32.85	4426.85	5	8	931.25			1248	9281	0.6	12769	30843	3619	13
25	50.85	7050.95	5	10	1707			1138	7318	0.6	17541	32861	7822	15
21	44.55	7203.74	7	13	2735.2			4349	9835	0.4	39292	57780	13739	16
14	36.23	7587.88	6	9	2155.6			4007	8674	0.6	59069	77015	12459	16
14	25.2	7939.62	6	14	1550.8			2111	8580	0.4	27373	57281	6730	20
21	44.55	6470.94	6	8	983.25			2212	5506	0.4	31400	50534	23628	8
27	58.05	8832.24	6	8	1547.8			1808	4961	0.8	21617	29243	8600	13
17	38.48	7519.74	8	10	1676.2			1748	4100	0.4	42076	55287	18280	15
15	22.5	4928.34	5	10	2546.6			1544	9443	0.6	20802	36371	7545	15
23	52.65	7046.49	5	11	1834.24			1238	4288	0.2	13334	22886	5234	13

Speed of the longest stroke (mm/s)	Strokes per minute (n)	Average drawing speed (mm/s)	Whole area (mm2)	long strokes(n)	short strokes(n)	Average element latency (ms)		dGCF 20-min delayed recall:	Transition time (ms)	Elapsed time of 5 early long strokes (ms)	First 5 stroke ratios (%)	Time in air (ms)	Total time (ms)	First stroke latency (ms)
63.73	20	48.38	7425.27	8	7	3177.4			0	5578	1	21930	34498	6001
93.09	33	57.38	5656.89	6	7	578.2			2014	6710	0.4	15730	27132	7743
46.25	24	49.27	6354.15	6	7	1087.2			0	5507	1	17278	28986	10233
211.25	30	86.85	9165.05	8	8	1753.4			2228	4434	1	19975	33461	5776
41.33	20	51.52	7579.58	7	6	666.8			709	8216	0.8	21178	34741	12410
61.68	30	44.55	3991.68	5	11	1120			1122	6356	0.6	23670	38056	6170
37.74	21	39.83	7310.4	7	7	921			3789	6334	1	19997	37568	4948
43.94	22	52.65	5613.3	7	7	1472.4			1873	6446	1	17825	32099	4415
52.85	14	42.3	6191.64	3	12	3936.8			4389	7064	0.6	32016	42027	13170
23.02	15	26.55	5545.72	7	8	2325.4			1547	10893	0.6	29012	53365	10019
38.07	20	32.63	4945.86	6	9	3083.75			1023	8242	0.8	12346	33702	5220
50.96	24	36.9	5766.19	5	12	1320.2			1043	7357	0.8	19886	36090	7873
53.32	21	36.9	4703.87	5	13	3354.8			1264	7126	0.6	17262	32520	5936
56.31	22	56.02	6930.36	7	9	806.6			993	9631	0.6	22468	42516	5464
42.83	16	35.77	6356.48	6	6	1325.75			0	10083	1	29050	50895	10849
27.12	15	27.23	6850.78	6	9	2713.8			7912	8738	0.8	35562	57614	6582
53.78	21	39.15	4175.35	6	8	1883.4			0	7221	1	13279	24122	4099
55.55	29	39.83	3103.72	3	14	1179.2			565	1019	0.2	13748	24647	2354
42.16	21	39.83	7148.6	6	8	1743.2			1400	8910	0.8	16871	31320	5756
17.01	18	20.7	4212	4	11	1372.4			8246	15335	0.4	22782	48055	4976
95.53	35	69.08	5070.85	6	9	909			0	5988	1	13228	24812	5568
104.57	33	68.17	4801.68	5	9	1046.2			1503	3304	0.6	13025	22076	5894
34.53	19	29.25	6040.27	6	9	1209.6			7575	10340	0.8	22399	50329	7257
99.79	29	65.25	8404.91	7	8	981.6			0	4621	1	17719	30291	5626
44.84	31	30.15	2227.5	1	17	1280			651	1859	0.2	17916	34543	4581
70.99	22	51.3	8464.5	8	7	1405.2			2937	9680	0.6	25671	40914	8548
78.96	17	41.63	5867.34	6	13	6391.75			4051	10736	0.4	39964	61328	11774
37.21	14	31.28	5915.43	4	14	7166.25			6065	12356	0.4	37893	61374	8419
46.95	21	42.75	6015.06	6	10	1433.6			1911	8978	0.6	23494	40500	6281
59.33	21	45.23	7208.8	6	10	1604.2			2110	5312	0.6	25467	43316	5904
43.79	13	34.43	8754.48	6	11	3149.6			7336	14394	0.8	39989	68228	11129
31.24	24	22.5	3300.24	2	18	1446.2			3418	4189	0	37840	62303	5944

66.33	26	37.13	6183.29	5	14	1591.4			2332	6546	0.4	40107	62239	7057
121.39	29	61.88	7252.74	7	13	2135.667			138	5831	0.8	15805	25678	8988
70.77	25	42.75	2863.5	2	12	3652.667			0	4099	1	23634	32601	13277
52.45	29	76.95	8556.94	6	9	2488.4			637	8490	0.6	25704	39884	9808
43.86	25	39.15	4256.75	6	10	1237.75			5583	9201	0.4	23244	38000	8124
25.93	22	32.63	4860.96	6	8	1230.2			1327	10198	0.6	24563	40090	3641
45.63	20	46.35	4546.88	5	5	1581.5			1197	8409	0.6	18350	33209	9877
53.69	25	38.7	5237.61	6	9	1056			1049	6066	1	19682	36449	5664
59.41	19	51.98	8282.25	7	6	2426.6			876	6566	0.8	21680	38355	6314
49.5	16	40.05	6349.59	6	6	3152.75			2111	9281	0.6	19723	34834	7293
38.63	20	49.5	5037.09	7	5	3614			1536	6466	0.6	13060	23340	7215
101.74	17	67.95	2977.36	5	5	4926.667			1082	4677	0.6	17244	25270	9152
41.99	19	51.75	11067.84	8	8	2590.75			5927	13777	0.6	27072	51638	8939
61.44	22	51.08	10037.67	9	7	1575			5830	10601	0.6	36546	58955	8623
86.45	15	44.55	8707.5	9	8	2902.8			3890	6378	0.8	34071	50466	8790
26.38	20	28.57	7291.01	7	10	1244.4			1019	12933	0.8	22589	50668	6851
30.03	18	21.38	2977.05	3	12	3045.6			40708	11732	0.4	65950	93659	6718
44.49	25	37.58	3484.72	4	7	952.6667			1583	4776	0.4	32314	50442	4280
43.97	13	29.7	3923.74	4	10	7851.667			2888	9393	0.4	46006	64463	8345
30.53	11	27.23	3381.95	5	11	3769			664	8830	0.8	47013	63423	11677
35.97	12	36.23	6327.72	8	2	5391.333			6404	9516	0.8	21992	38328	5768
57.51	23	30.6	2961.56	4	12	1155			1876	4656	0.4	19036	36288	7520
37.7	25	31.95	3933.56	5	8	981.5			1139	9917	0.6	15147	34807	4918
47.95	27	42.52	4643.73	5	10	1029.4			1006	8366	0.6	18312	35580	8811
59.73	17	37.58	4360.33	5	11	2282.8			1534	8678	0.8	23638	40818	7175
50.07	12	36	4622.77	6	10	10417.33			3184	7535	0.4	39510	55289	18367
30.47	221	22.28	3876.36	3	17	1392.25			2775	10056	0.6	24236	49305	12418
53.57	12	55.8	5852.2	4	4	1845.4			12583	5766	0	35058	53814	11938
81.36	27	106.88	7808.5	7	6	1664.6			3412	5769	0.8	14090	23202	5532
67.12	16	37.13	3997.2	3	12	4152.5			1393	3566	0.6	29732	39624	19355
46.33	25	36	4331.27	5	10	1598			1371	8310	0.6	16493	30964	5006
47.66	34	51.75	4433.74	4	9	1062			1729	3625	0	16801	27635	7710

total strokes (n)	Speed of the longest stroke (mm/s)	Strokes per minute (n)	Strokes per minute (n)	Whole area (mm2)	long strokes(n)	short strokes(n)	Average element latency (ms)	
15	64.02	26	61.65	7320.98	7	8	1760.2	
12	62.94	27	58.73	5420.62	5	7	709.2	
17	99.63	27	69.08	7640.38	7	10	2314.2	
14	110.69	25	69.3	10639.15	7	7	1735.2	
13	53.05	22	57.15	7541.2	7	6	680.2	
17	59.78	27	41.63	3873.02	5	12	1346.6	
14	46.64	22	49.5	8573.04	8	6	921.12	
13	49.24	24	56.93	7118.69	8	5	1129.2	
15	38.43	21	57.83	5363.06	5	10	2017	
15	25.64	19	32.18	5425.18	6	9	1514.4	
15	60.12	27	32.63	4945.86	6	9	579.1	
17	58.19	28	45	6268.54	6	11	933.2	
16	71.18	30	45.69	5204.66	6	10	861.6	
17	39.83	24	40.5	7358.85	7	10	1667.6	
13	42.85	15	35.77	6418.74	7	6	1438.25	
15	64.19	16	35.1	6738.39	8	7	2644	
11	71.55	27	53.33	6126.79	5	6	852	
16	67.53	39	37.35	1933.88	1	15	940.4	
14	51.61	27	41.85	5997.54	6	8	1182.8	
15	27.72	19	22.95	4647.38	5	10	982.8	
14	104.88	34	56.02	4761.43	5	9	770.75	
14	107.54	38	69.53	4787.71	4	10	620	
16	46.2	19	33.98	8135.49	9	7	1453	
16	107.98	32	66.15	8365.07	7	9	1023.6	
15	40.03	26	26.32	2296.96	1	14	664.6	
14	62.93	21	47.7	6290.06	6	8	1203.2	
17	30.4	17	33.52	5972.48	7	10	3686.25	
18	85.09	18	30.82	6299.02	8	10	3196.5	
16	50.78	24	37.35	4827.6	5	11	1149.4	
20	47.01	28	39.38	5263.38	4	16	1344.8	
18	44.95	16	36	9602.75	8	10	2394.2	
20	26.77	19	25.2	4096.57	2	18	2689	

24	60.21	23	37.35	7014.25	4	20	2006	
14	79.52	33	70.42	5438.34	7	7	1009.333	
10	100.36	18	71.33	5197.87	8	2	5731	
15	42.91	23	57.6	10256.32	5	10	1642	
16	43.86	21	39.15	4256.75	6	10	1624.2	
14	28.21	30	41.4	5437.88	6	8	671	
11	46.36	20	41.85	4947.18	5	6	1278.5	
15	57.91	25	45	5779.5	8	7	1427.25	
16	54.34	25	48.6	7214.27	7	9	1720.6	
12	52.01	21	46.35	5751.96	6	6	1624	
8	34.89	21	45.76	7652.68	6	2	910	
11	96.83	27	71.33	3568.46	5	6	1406.667	
16	30.27	19	40.95	9976.77	7	9	2126.6	
18	53.99	18	42.3	9816.9	8	10	3349.5	
16	85.83	19	58.73	9267.56	8	8	3048.8	
16	26.54	19	28.13	6958.91	7	9	1376.4	
17	24.46	11	18.9	2997.1	4	13	2352.2	
12	33.47	14	24.07	2426.96	2	10	4012	
14	43.97	13	29.7	3923.74	4	10	2218	
16	48.5	15	36.45	3983.68	5	11	5816.5	
11	46.62	17	39.6	5309.55	8	3	1630.667	
15	44.42	25	32.4	2904.61	3	12	1102.25	
13	34.38	22	33.98	5127.91	5	8	1406.5	
15	38.25	25	33.75	3773.39	5	10	975.8	
14	70.24	21	41.4	5371.11	8	6	1723.75	
13	38.83	14	36	5203.44	4	9	3335.667	
16	35.5	19	27	4159.35	4	12	938.25	
9	62.22	12	42.75	6875.43	3	6	1734.5	
11	73.52	28	84.83	7755.04	7	4	811.2	
14	99.42	21	51.3	4742.04	4	10	1335.25	
15	44.55	29	37.8	4510.79	5	10	1326.8	
14	49.72	30	43.2	3590.93	3	11	991	
